# PSMA-targeted fluorescent probe for NIR-II imaging in prostate cancer intraoperative navigation and tumor margin mapping

**DOI:** 10.7150/thno.117540

**Published:** 2026-02-11

**Authors:** Zhongji Jiang, Haozhe Tan, Bo Wu, Lin Zhang, Gaohaer Kadeerhan, Jin Zhang, Jiali Jin, Zikuan Zhang, Hong Guo, Wenmin Guo, Jiedong Jia, Jun Tian, Ben Zhong Tang, Dongwen Wang

**Affiliations:** 1 Department of Biology, School of Medicine, Southern University of Science and Technology, Shenzhen, Guangdong Province, 518055, China.; 2 Department of Urology, National Cancer Center/National Clinical Research Center for Cancer/Cancer Hospital & Shenzhen Hospital, Chinese Academy of Medical Sciences and Peking Union Medical College, Shenzhen, Guangdong Province, 518116, China.; 3 Guangdong Basic Research Center of Excellence for Aggregate Science, School of Science and Engineering, The Chinese University of Hong Kong (Shenzhen), Shenzhen, Guangdong Province, 518172, China.; 4 Department of Urology, First Hospital of Shanxi Medical University, Taiyuan, Shanxi Province, 030001, China.; 5 Central Laboratory & Shenzhen Key Laboratory of Epigenetics and Precision Medicine for Cancers, National Cancer Center/National Clinical Research Center for Cancer/Cancer Hospital and Shenzhen Hospital, Chinese Academy of Medical Sciences and Peking Union Medical College, Shenzhen, Guangdong Province, 518116, China.; 6 School of Medicine, Southern University of Science and Technology, Shenzhen, Guangdong Province, 518116, China.

**Keywords:** prostate cancer, prostate-specific membrane antigen (PSMA), near-infrared fluorescence imaging, albumin binding, intraoperative navigation, tumor margin mapping

## Abstract

Accurate delineation of tumor margins during prostate cancer surgery remains challenging due to limited intraoperative visualization and insufficient molecular specificity. Here, we developed a PSMA-targeted near-infrared fluorescent probe, PSMA-12-IRDye800CW, that leverages the clinically used IRDye800CW scaffold and its extended emission tail beyond 1000 nm to support NIR-II fluorescence imaging for intraoperative navigation and histopathological margin mapping.

**Methods:**

PSMA-12-IRDye800CW integrates a PSMA-targeting ligand with an albumin-binding linker to enable active targeting and circulation-assisted tumor accumulation. Optical properties, targeting specificity, imaging performance, and biosafety were evaluated *in vitro*, in prostate cancer xenograft models with direct comparison to indocyanine green (ICG) across defined time points, and in clinical formalin-fixed paraffin-embedded (FFPE) prostate specimens with matched histopathology and PSMA immunohistochemistry.

**Results:**

In 22Rv1 (PSMA⁺) xenografts, PSMA-12-IRDye800CW achieved significantly higher tumor-to-background ratios than ICG at key surgical-relevant time points, including 24 h (4.31 ± 0.17 vs. 2.65 ± 0.15), providing a practical imaging window for fluorescence-guided resection. *Ex vivo* tissue analyses further confirmed significantly higher fluorescence in tumors than in muscle and skin. In human FFPE specimens, fluorescence showed pathology-aligned spatial correspondence with PSMA immunohistochemistry, and fluorescence intensity correlated strongly with PSMA H-scores (R² = 0.8616, P < 0.0001), enabling micron-scale histopathological margin mapping. Multimodal biosafety assessments indicated favorable biocompatibility with no evident acute toxicity and low immunogenic potential.

**Conclusions:**

PSMA-12-IRDye800CW enables NIR-II fluorescence imaging-assisted intraoperative navigation and provides a quantitative, pathology-anchored readout for histopathological margin mapping in prostate cancer, supporting further clinical validation of this PSMA-targeted strategy for fluorescence-guided surgery and margin assessment.

## Introduction

Prostate cancer (PCa) is among the most common malignancies and remains a leading cause of cancer-related mortality in men worldwide 1, 2. The Lancet Commission projects that global annual PCa cases will nearly double by 2040, accompanied by an 85% increase in deaths over the same period 3. Despite therapeutic advances, achieving complete tumor resection remains challenging due to the prostate's complex anatomy and the frequent occurrence of regional lymph node involvement 4-7. These factors contribute to positive surgical margins and disease recurrence, underscoring the need for improved intraoperative guidance and complementary post-surgical pathological assessment 8, 9. Such integrated strategies are essential for accurate margin delineation and improved long-term oncological outcomes.

Radical prostatectomy remains a cornerstone of PCa management, yet intraoperative decisions still largely rely on white-light inspection of macroscopic features, which is insufficient to identify microscopic tumor boundaries or small occult lesions 4, 10, 11. Fluorescence-guided surgery (FGS) has therefore emerged as an enabling strategy for real-time intraoperative visualization, particularly in near-infrared (NIR) imaging. Beyond the conventional NIR-I range (∼700-900 nm), fluorescence detection in the > 1000 nm (NIR-II window; 1000-3000 nm) can reduce photon scattering and tissue autofluorescence, thereby improving imaging depth and contrast and supporting more informative surgical navigation 12-14. Although NIR-I imaging is clinically established (e.g., vascular imaging and tumor detection) 15, 16, and several targeted NIR-I agents are under clinical evaluation 17, 18, there remains a need for molecularly targeted probes and standardized quantitative readouts that directly inform tumor boundary assessment during and after surgery 19-22.

Prostate-specific membrane antigen (PSMA) is a type II transmembrane glycoprotein substantially upregulated in PCa and associated with aggressive disease features, supporting its use as a clinically relevant biomarker and target 23-26. Structurally, PSMA contains a funnel-shaped binding cavity with S1 and S1' subsites that enables rational ligand design 27, 28. PSMA-targeted modalities, including PSMA Positron Emission Tomography (PET) imaging and PSMA-directed radioligand therapy (RLT), have become integral to clinical staging and management by enabling sensitive lesion localization 8, 27-29. In parallel, albumin-binding strategies have been widely explored to prolong systemic circulation and improve tumor-associated exposure of small-molecule agents, thereby expanding practical imaging windows; however, the contribution of enhanced permeability and retention (EPR)-mediated accumulation can vary across tumor types and models and should be interpreted cautiously 30, 31.

Several studies have demonstrated the promising potential of PSMA-targeted NIR fluorescence agents for guiding surgery in PCa. For example, YC-27 was developed as a low-molecular-weight urea-based NIR fluorophore, targeting PSMA to facilitate intraoperative imaging of prostate tumors. It has been shown to improve tumor detection and reduce positive surgical margins during laparoscopic resection in preclinical models 32-35. Further refinement of such agents, including OTL78, has resulted in optimized NIR fluorescence for enhanced real-time tumor detection and surgical guidance 36, 37. In a phase 2a clinical trial, OTL78 demonstrated excellent tumor-to-background contrast and was well-tolerated in patients undergoing robot-assisted radical prostatectomy 18. Similarly, ^68^Ga-P3, a dual-modality probe, has been successfully used for PET/CT imaging and FGS in PCa, offering a comprehensive approach for tumor detection both preoperatively and intraoperatively 38. However, the majority of these studies focus on the NIR-I fluorescence window. To overcome its limitations, we need to achieve deeper wavelength NIR-II imaging, which holds promise for clinical applications.

In this study, we engineered a PSMA-targeted small-molecule probe by introducing an albumin-binding moiety and conjugating IRDye800CW, a clinically used near-infrared dye with favorable aqueous solubility and biosafety that enables NIR-II imaging when acquired with a 1000 nm long-pass emission filter. The resulting probe, PSMA-12-IRDye800CW, integrates PSMA-mediated active targeting with albumin-assisted prolonged circulation to support a practical time window for intraoperative imaging. Motivated by the limitations of current PSMA-targeted fluorophores and the need for stronger surgery-to-pathology evidence chains, we designed this work to address three testable questions: (i) whether NIR-II imaging provides measurable gains in resolution and contrast compared with NIR-I under matched conditions; (ii) whether the PSMA-targeting design offers a time window suitable for intraoperative navigation with quantitative tumor-to-background readouts; and (iii) whether fluorescence patterns in clinical formalin-fixed paraffin-embedded (FFPE) specimens spatially correspond to PSMA immunohistochemistry (IHC) to support micron-scale histopathological margin mapping. Collectively, our study presents the design, synthesis, and validation of PSMA-12-IRDye800CW and supports its potential for intraoperative navigation and pathology-aligned margin assessment in PCa.

## Methods

### Prostate cancer atlas and clinical patient cohort

Bulk RNA-sequencing data were retrieved from the Prostate Cancer Atlas (https://prostatecanceratlas.org) 39, comprising normal prostate tissues (n = 173), primary PCa (n = 708), and castration-resistant PCa (CRPC, n = 484), including neuroendocrine PCa (NEPC, n = 34) and double-negative PCa (DNPC, n = 22) subtypes. Principal component analysis (PCA) and PSMA (*FOLH1*) expression quantification were performed to characterize molecular progression.

Additionally, ten FFPE PCa surgical specimens were collected for IHC validation. All specimens were independently confirmed by two pathologists. Ethical approval was obtained from the Ethics Committee of Shenzhen Hospital, Cancer Institute and Hospital, Chinese Academy of Medical Sciences (No. JS2024-7-1).

### Cell culture and flow cytometry for PSMA expression analysis

The human prostate cell lines 22Rv1, PC-3, LNCaP, VCaP, and RWPE-1 were obtained from the Shanghai Cell Bank (Chinese Academy of Sciences, Shanghai, China) and authenticated by short tandem repeat (STR) profiling. 22Rv1, PC-3, and LNCaP cells were cultured in RPMI-1640 medium; VCaP cells in high-glucose DMEM; and RWPE-1 cells in Keratinocyte-SFM medium. All media were supplemented with appropriate growth factors and 1% penicillin-streptomycin. Cells were maintained at 37 °C in a humidified incubator with 5% CO₂.

For flow cytometry analysis, cells were harvested, resuspended as single-cell suspensions, and incubated with fluorophore-conjugated anti-PSMA antibodies for 30 minutes at room temperature in the dark. Samples were analyzed using a flow cytometer. All experiments were performed in triplicate.

### Confocal microscopy for PSMA-dependent cellular association of PSMA-12-FITC

To visualize PSMA-dependent cellular association at the microscopic level, PSMA-12-FITC was prepared by replacing IRDye800CW with fluorescein isothiocyanate (FITC) while retaining the same PSMA-12 ligand scaffold. LNCaP (PSMA⁺) and PC-3 (PSMA⁻) cells were seeded onto poly-L-lysine-coated glass-bottom dishes and cultured overnight. Cells were then incubated with PSMA-12-FITC (20 μg/mL) in serum-free medium at 37 °C for 30 min. After incubation, cells were washed three times with PBS to remove unbound probe, fixed with 4% paraformaldehyde, and counterstained with DAPI. Confocal fluorescence imaging was performed using a laser scanning confocal microscope (Leica, Germany). Acquisition settings were kept constant between groups within each experiment. All experiments were independently repeated three times.

### Establishment of subcutaneous xenograft tumor models

Male BALB/c nude mice (4-6 weeks old) were subcutaneously injected with 1 × 10⁷ 22Rv1 (PSMA⁺) cells suspended in PBS containing 50% Matrigel (Corning, USA). Tumor growth was monitored regularly, and mice with tumor volumes reaching approximately 200 mm³ were selected for *in vivo* imaging. All animal procedures were conducted in accordance with institutional guidelines and approved by the Institutional Animal Care and Use Committee (Approval No. TOPGM-IACUC-2023-0111).

### Evaluation of imaging depth in tissue-mimicking models

All fluorescence imaging was conducted under standardized conditions (808 nm excitation, 20 mW/cm²; 50 ms exposure; fixed camera gain). NIR-I images were collected with a silicon-based detector using an 850 nm long-pass filter, and NIR-II images were collected with an InGaAs detector using a 1000 nm long-pass filter, under identical excitation and acquisition settings unless stated otherwise. For depth-penetration studies, PSMA-12-IRDye800CW (200 μg/mL) was loaded into capillary tubes and overlaid with chicken breast tissue layers (0-8 mm). Depth-dependent line profiles were extracted from identical regions (dashed lines) using ImageJ, and structural separability was evaluated by comparing line-profile peak definition across depths. Spatial resolvability at shallow depth was quantified by Gaussian fitting and reported as full width at half maximum (FWHM).

### *In vivo* NIR-II fluorescence imaging and fluorescence-guided surgery

Male mice bearing 22Rv1 xenografts were intravenously injected with 200 μL of a 200 μg/mL solution of PSMA-12-IRDye800CW or indocyanine green (ICG). Serial NIR-II imaging was performed at 1, 3, 6, 12, 24, and 48 h post-injection under standardized imaging settings (808 nm excitation, 20 mW/cm²; 50 ms exposure; 1000 nm long-pass emission filter). During imaging, mice were anesthetized and placed on a temperature-controlled stage.

At 48 h post-injection, real-time NIR-II FGS was performed to delineate tumor-associated fluorescence, resect tumors, and identify fluorescence-positive lymph nodes when present. Resected specimens were collected for pathological validation using Hematoxylin and Eosin (H&E) staining and PSMA IHC. Regions of interest (ROIs) were placed over tumors and a background region (adjacent normal tissue or contralateral muscle). The tumor-to-background ratio (TBR) was calculated as the ratio of mean fluorescence intensity (MFI) in the tumor to that in the background (MFI_tumor/MFI_background).

### NIR-II fluorescence imaging and PSMA-aligned mapping in clinical FFPE specimens

FFPE sections (5 μm) from PCa patients were deparaffinized, subjected to antigen retrieval, and blocked with serum. Sections were incubated with PSMA-12-IRDye800CW (20 μg/mL) overnight at 4 °C, washed with PBS, and imaged under standardized NIR-II settings. Fluorescence intensity within tumor and adjacent regions was quantified by MFI and TBR. Fluorescence imaging and PSMA IHC were performed on adjacent serial sections from the same FFPE block. Adjacent serial sections were processed for PSMA IHC, DAPI staining, and H&E staining for spatial correspondence assessment.

### Biosafety evaluation and basophil activation test (BAT)

The biosafety of PSMA-12-IRDye800CW was assessed through histopathological, biochemical, and immunological analyses. Mice received intravenous injections of PSMA-12-IRDye800CW (2 mg/mL), IRDye800CW, or PBS. Blood samples were collected at 72 hours post-injection to measure serum blood urea nitrogen (BUN), alanine aminotransferase (ALT), and aspartate aminotransferase (AST) levels. Mouse body weight was monitored every two days for 21 days. At the endpoint, major organs (heart, liver, spleen, lung, kidney) were harvested and subjected to H&E staining to assess pathological changes.

To evaluate immunological safety, BAT was performed using peripheral blood collected from healthy donors. PSMA-12-IRDye800CW was prepared at a final concentration of 200 μg/mL and incubated with donor whole blood in the presence of stimulation buffer. Basophils were stained with anti-CCR3-PE and anti-CD63-PE-Dy647 antibodies, followed by flow cytometric analysis. The stimulation index (SI) was calculated as SI = Avg/Background based on flow cytometric readouts, with an SI ≥ 2 indicating potential hypersensitivity 37.

### Quantitative analysis and statistical methods

IHC staining was quantified by H-score, integrating staining intensity and the percentage of positive cells; digital quantification was assisted by ImageJ (IHC Profiler) when applicable. Excitation/emission spectra were normalized and fitted in OriginPro 2018C. Capillary width was determined by Gaussian fitting and reported as FWHM. NIR fluorescence images were pseudocolored (“Gem” LUT) and analyzed in ImageJ. Data are presented as mean ± SD. Two-group comparisons were performed using unpaired two-tailed t-tests, whereas comparisons among three or more groups were performed using one-way ANOVA with appropriate post hoc tests (GraphPad Prism 9). Statistical significance was defined as *P < 0.05, **P < 0.01, ***P < 0.001, and ****P < 0.0001.

## Results

### PSMA expression is upregulated across PCa progression

**Figure 1:**
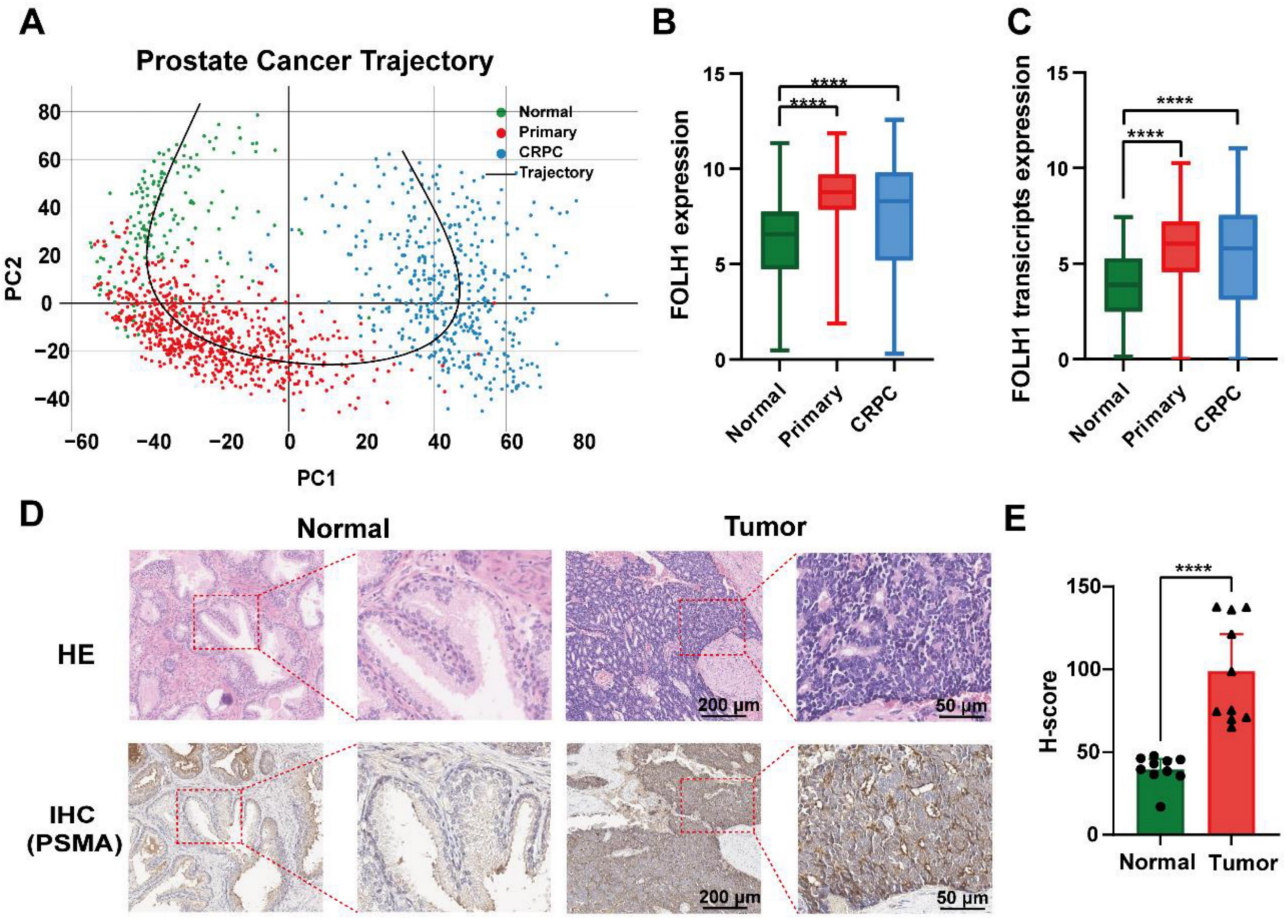
** Dynamic PSMA (*FOLH1*) expression across PCa progression.** (A) Principal component analysis (PCA) illustrating the transcriptomic trajectory from normal prostate tissues (green, n = 173) to primary PCa (red, n = 708) and CRPC (blue, n = 484). (B) Boxplot showing gene-level *FOLH1* expression in bulk tissue across normal, primary PCa, and CRPC cohorts. (C) Boxplot showing transcript-level *FOLH1* expression across the same cohorts. (D) Representative H&E and PSMA IHC images of tumor versus adjacent normal tissues. (E) Quantitative H-score analysis showing significantly elevated PSMA expression in tumor tissues (****P < 0.0001).

Transcriptomic analysis of bulk RNA-sequencing data from normal prostate tissues, primary PCa, and CRPC revealed a progressive shift along the disease continuum (Fig. 1A) accompanied by significantly increased PSMA (*FOLH1*) expression in primary PCa and bulk CRPC relative to normal tissues at both the gene and transcript levels (Fig. 1B-C, P < 0.0001; Table S1). Notably, subtype-resolved analyses further indicated reduced *FOLH1* expression in NEPC and DNPC compared with the overall CRPC cohort (Fig. S1A-B, P < 0.0001), underscoring molecular heterogeneity in advanced disease. Protein-level validation in an independent clinical FFPE cohort (n = 10; clinicopathological characteristics summarized in Table S2) confirmed significantly higher PSMA expression in tumor regions than in adjacent normal tissues by IHC (Fig. 1D-E; tumor H-score: 98.82 ± 31.22 vs. adjacent H-score: 39.39 ± 8.94, P < 0.0001). Collectively, these data support PSMA as a clinically relevant molecular target in PSMA-high PCa and establish a clinical rationale for PSMA-directed fluorescence imaging strategies toward intraoperative navigation and pathology-aligned margin assessment.

### Structural design and optical characterization of PSMA-12-IRDye800CW supporting NIR-II imaging

**Figure 2:**
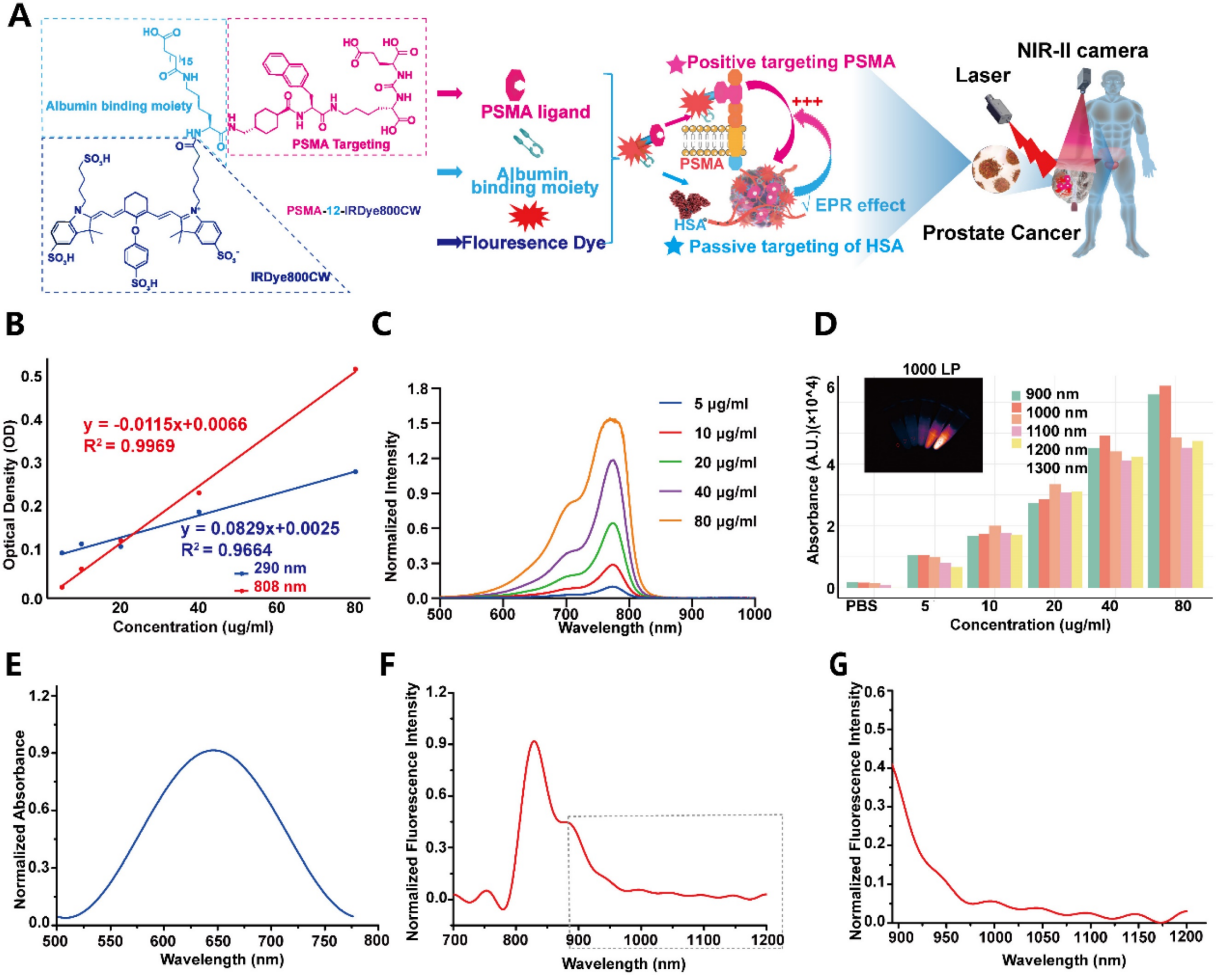
** Optical characterization of PSMA-12-IRDye800CW.** (A) Chemical structure of PSMA-12-IRDye800CW and schematic illustration of its PSMA-targeting design for fluorescence imaging, including NIR-II imaging capability under a 1000 nm long-pass detection window. (B) Standard calibration curves obtained from optical density measurements at 290 nm and 808 nm. (C) UV-vis absorption spectra of PSMA-12-IRDye800CW at increasing concentrations. (D) Linear correlation between probe concentration and fluorescence intensity, supporting quantitative detection. (E) Normalized absorbance spectrum of PSMA-12-IRDye800CW. (F) Normalized fluorescence emission spectrum of PSMA-12-IRDye800CW. (G) Long-wavelength emission profile collected beyond 900 nm, with NIR-II-relevant signals evaluated under 1000 nm detection conditions.

We synthesized PSMA-12-IRDye800CW as a PSMA-targeted fluorescent probe incorporating an albumin-interaction linker to support prolonged circulation and tumor accumulation. The probe integrates three functional components: an ACUPA (Glu-urea-Lys) motif for PSMA binding, an α,ω-dicarboxylic acid linker (C18) designed for reversible albumin interaction, and IRDye800CW as the near-infrared fluorophore (Fig. 2A; Fig. S2). The key peptide intermediate PSMA-12 (Compound 6) was validated by its expected molecular weight (Fig. S3), high analytical purity by HPLC (98.53%; Fig. S4), and corroborating MALDI-TOF MS and ¹H/¹³C NMR spectra (Fig. S5-S7). The chemical structures and molecular weights of PSMA-12-IRDye800CW and the FITC-labelled analogue used for cellular experiments were further summarized in Fig. S8.

Optical calibration curves measured at 290 and 808 nm supported quantitative readouts and yielded a fluorophore-to-peptide (F/P) ratio of ~1.0 for PSMA-12-IRDye800CW (Fig. 2B). UV-vis absorption spectra showed a concentration-dependent increase with a characteristic absorption maximum at 774 nm (Fig. 2C), and fluorescence intensity displayed a linear correlation with probe concentration, supporting quantitative fluorescence detection (Fig. 2D). Normalized absorbance and emission spectra further revealed a dominant fluorescence emission peak at 829 nm (Fig. 2E-F), together with a measurable long-wavelength tail beyond 900 nm (Fig. 2G). This extended emission enables NIR-II imaging when fluorescence is collected using a 1000 nm long-pass emission filter (Fig. 2G).

### Phantom validation of NIR-I versus NIR-II imaging performance

**Figure 3:**
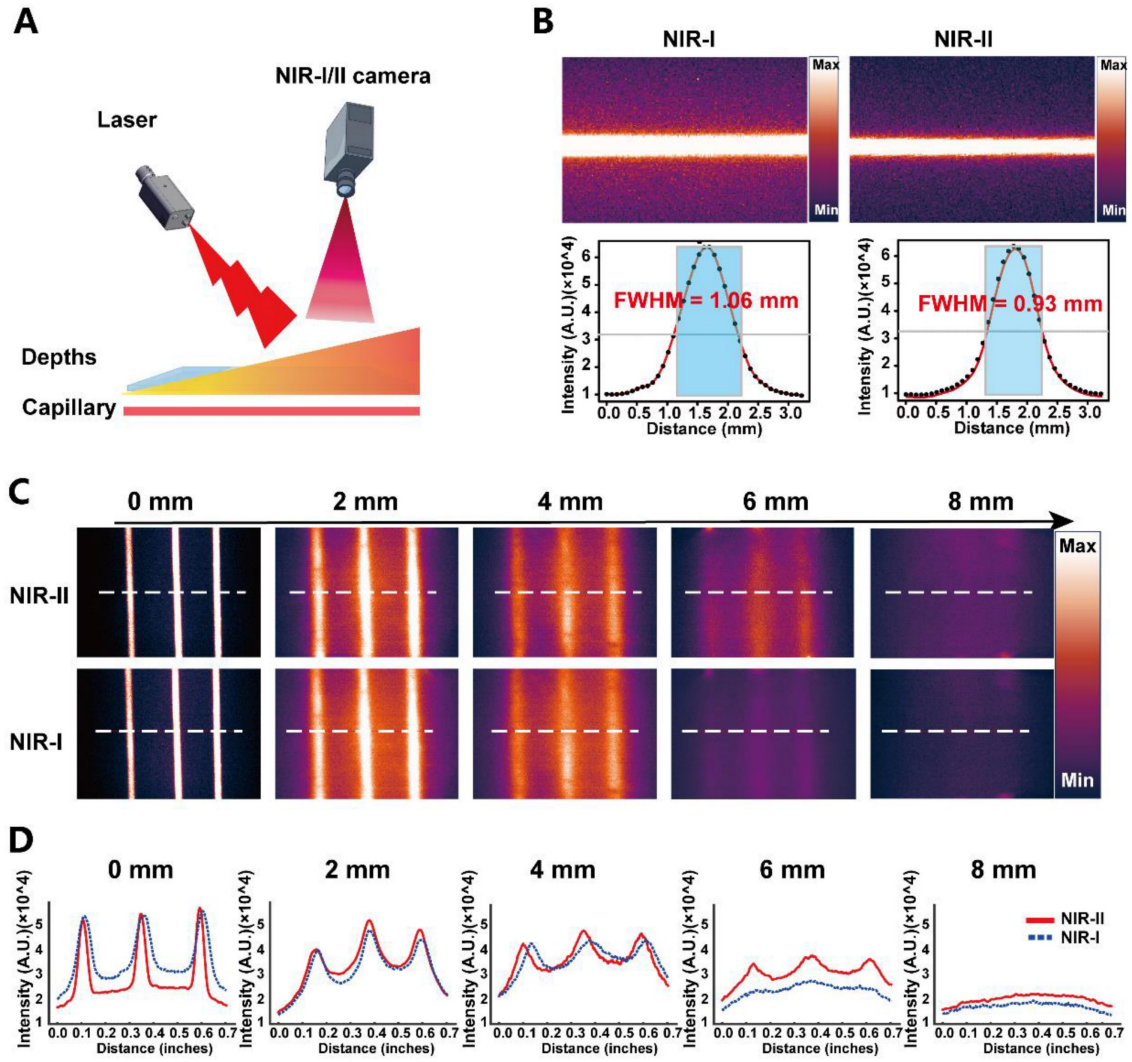
** Phantom comparison of NIR-I and NIR-II imaging performance of PSMA-12-IRDye800CW.** (A) Schematic of the tissue-phantom setup. Capillary tubes containing PSMA-12-IRDye800CW were covered with chicken breast tissue of increasing thickness and imaged under NIR-I or NIR-II acquisition. (B) Representative NIR-I (left) and NIR-II (right) images with line profiles and Gaussian fitting, showing improved spatial definition under NIR-II detection (FWHM: 1.06 mm vs. 0.93 mm). (C) Depth-dependent fluorescence images (0-8 mm) showing better preservation of capillary features under NIR-II imaging. Dashed lines indicate regions used for profile extraction. (D) Corresponding line profiles across depths, demonstrating improved peak separability under NIR-II imaging. Imaging was performed under matched conditions with 808 nm excitation; NIR-II signals were collected using a 1000 nm long-pass filter.

To benchmark imaging performance under tissue-mimicking scattering conditions, we constructed a capillary-tube phantom in which PSMA-12-IRDye800CW-filled capillaries were imaged through increasing thicknesses of chicken breast tissue (0-8 mm) using NIR-I and NIR-II acquisition settings (Fig. 3A) 21. Under matched conditions, the representative images in Fig. 3B (NIR-I, left; NIR-II, right) illustrate finer structural definition under NIR-II imaging, which was further quantified by a narrower Gaussian-derived FWHM (0.93 mm vs. 1.06 mm). As tissue thickness increased, fluorescence signals attenuated in both channels; however, NIR-II imaging more effectively preserved the visibility and separability of capillary features at greater depths (Fig. 3C). Line-profile analyses corroborated this trend by showing better maintenance of peak structure and spatial features for NIR-II compared with NIR-I across depth increments (Fig. 3D). Together, these phantom results support that PSMA-12-IRDye800CW enables NIR-II imaging with improved spatial definition and more robust depth performance relative to NIR-I in tissue-mimicking settings.

### PSMA expression and cellular specificity of PSMA-12-IRDye800CW in prostate cell lines

**Figure 4:**
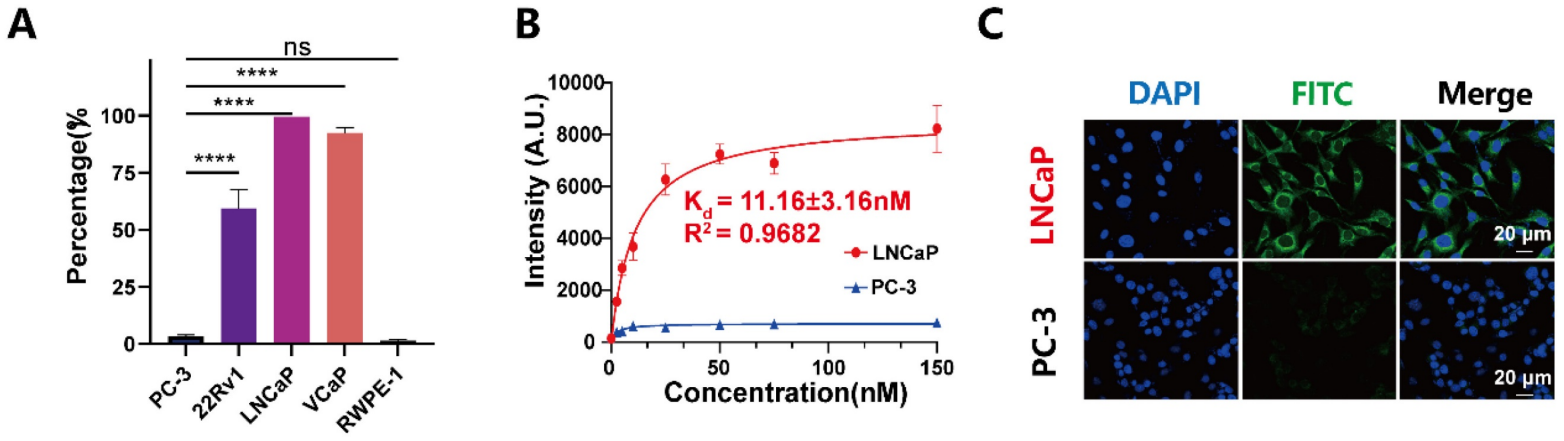
** PSMA-12-IRDye800CW enables specific imaging of PSMA-positive prostate cancer cells.** (A) Flow cytometry analysis of PSMA expression in PC-3, 22Rv1, LNCaP, VCaP, and RWPE-1 cells, confirming differential receptor levels across cell lines. (B) Binding affinity of PSMA-12-IRDye800CW toward PSMA⁺ LNCaP cells compared with PSMA⁻ PC-3 cells, quantified by fluorescence spectrophotometry (Kd, dissociation constant). (C) Confocal fluorescence imaging of PSMA-12-FITC after incubation with LNCaP (PSMA⁺) or PC-3 (PSMA⁻) cells in serum-free medium at 37 °C for 30 min. Nuclei (blue), and PSMA-12-FITC (green). Images were acquired using identical settings across groups. Scale bar = 20 μm.

To define PSMA expression patterns and validate cellular targeting specificity, we profiled five prostate cell lines (PC-3, 22Rv1, LNCaP, VCaP and RWPE-1) by flow cytometry. PSMA expression was highest in LNCaP and VCaP cells, moderate in 22Rv1, and minimal to undetectable in PC-3 and RWPE-1, supporting the latter two as PSMA-low/negative controls 40 (Fig. 4A). Given their slower proliferation and limited tumorigenicity *in vivo*, LNCaP cells were prioritized for *in vitro* binding assays, whereas 22Rv1 cells, which combine robust PSMA expression with reliable xenograft formation, were used for subsequent *in vivo* studies 41, 42.

We next quantified the binding affinity of PSMA-12-IRDye800CW using fluorescence spectrophotometry. The probe exhibited high-affinity binding to PSMA⁺ LNCaP cells with a dissociation constant (Kd) of 11.16 ± 3.16 nM (R² = 0.9682), whereas binding to PSMA⁻ PC-3 cells remained negligible, indicating low nonspecific association under the tested conditions (Fig. 4B).

To visualize PSMA-dependent cellular association, we synthesized a fluorescein-labeled analogue (PSMA-12-FITC) by replacing IRDye800CW with FITC (Fig. S8). After incubation under physiological conditions (37 °C, 30 min), confocal microscopy revealed strong fluorescence in PSMA⁺ LNCaP cells but minimal signals in PSMA⁻ PC-3 cells (Fig. 4C). In LNCaP cells, fluorescence was primarily membrane-associated with partial intracellular signals, consistent with receptor-dependent binding and subsequent cellular internalization. Collectively, these results demonstrate high cellular selectivity of PSMA-12-based probes toward PSMA-positive PCa cells and support PSMA-mediated specificity for downstream *in vivo* imaging.

### *In vivo* NIR-II fluorescence imaging enables an operative window for intraoperative navigation and fluorescence-guided resection

**Figure 5:**
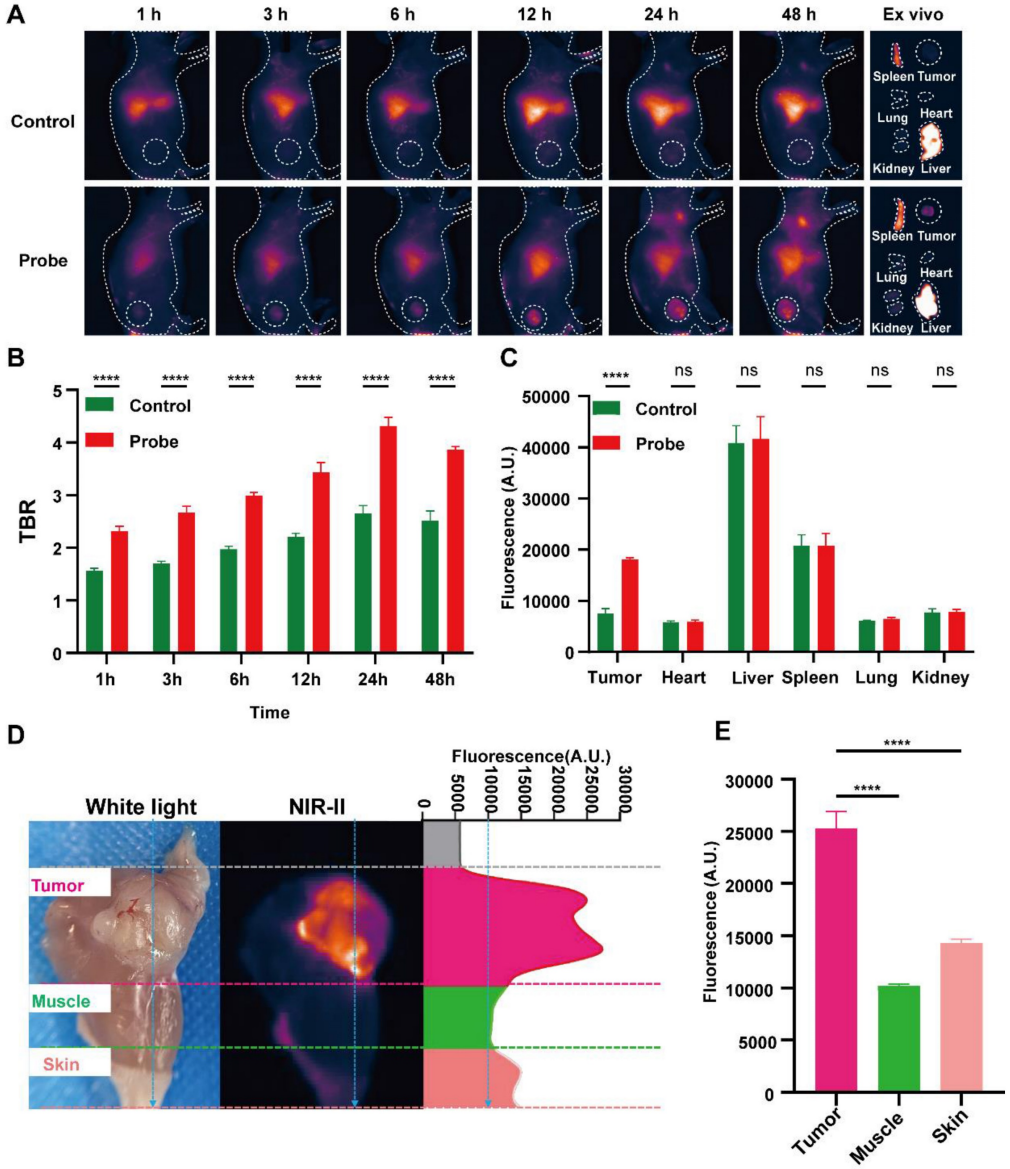
** PSMA-12-IRDye800CW enables tumor-specific imaging *in vivo*.** (A) NIR-II fluorescence imaging of 22Rv1 (PSMA⁺) tumor-bearing mice following intravenous injection of PSMA-12-IRDye800CW (Probe) or ICG, with corresponding *ex vivo* fluorescence images of major organs. (B) TBR quantified at 1, 3, 6, 12, 24, and 48 h post-injection. (C) Quantitative analysis of fluorescence intensity in tumors and major organs collected *ex vivo* at 48 h post-injection. (D) *Ex vivo* fluorescence comparison among tumor, muscle, and skin tissues at 48 h post-injection. (E) Quantitative fluorescence analysis showing significantly higher signal intensity in tumor tissues compared with muscle and skin. All imaging was performed using an 808 nm excitation laser (20 mW/cm²), 50 ms exposure time, and a 1000 nm long-pass emission filter (****P < 0.0001; ns, not significant).

Building on the optical performance and PSMA-dependent specificity, we assessed whether PSMA-12-IRDye800CW provides a practical operative imaging window for intraoperative navigation by performing time-resolved NIR-II imaging in 22Rv1 (PSMA⁺) xenograft-bearing mice with direct comparison to ICG (Fig. 5). Serial NIR-II imaging after intravenous injection showed progressive tumor signal enhancement and improved tumor-to-background contrast for PSMA-12-IRDye800CW compared with ICG (Fig. 5A-B). Quantitatively, at 24 h post-injection, the probe achieved a markedly higher TBR than ICG (4.31 ± 0.17 vs. 2.65 ± 0.15, respectively; Fig. 5B). *Ex vivo* imaging and biodistribution analysis at 48 h further supported preferential tumor signal enrichment for PSMA-12-IRDye800CW under matched imaging conditions (Fig. 5A, C). Importantly, *ex vivo* fluorescence comparison among tumor, muscle, and skin demonstrated that these tissue compartments could be reliably differentiated by NIR-II signals, with significantly higher fluorescence in tumors than in muscle and skin (Fig. 5D-E), supporting a practical contrast basis for margin-oriented resection. We next assessed intraoperative feasibility using NIR-II FGS (Movie 1; Fig. S9). Real-time NIR-II imaging enabled visualization of the primary tumor and guided resection, followed by inspection for fluorescence-positive residual foci and lymph nodes (Fig. S9). Given that fluorescence alone cannot distinguish passive retention or lymphatic trafficking from true metastasis, fluorescence-positive lymph nodes were not interpreted as malignant based solely on signal. Instead, all resected tissues were subjected to postoperative validation by H&E staining and PSMA IHC, enabling pathology-anchored interpretation of fluorescence findings (Fig. S9B-C). Together, these findings establish a quantified *in vivo* imaging window for navigation (e.g., 24 h TBR 4.31 ± 0.17) and demonstrate fluorescence-guided resection with pathology-anchored validation, supporting translation toward image-guided PCa surgery (Fig. 5; Fig. S9; Movie 1).

### Tumor margin mapping in clinical PCa specimens using NIR-II fluorescence imaging

**Figure 6:**
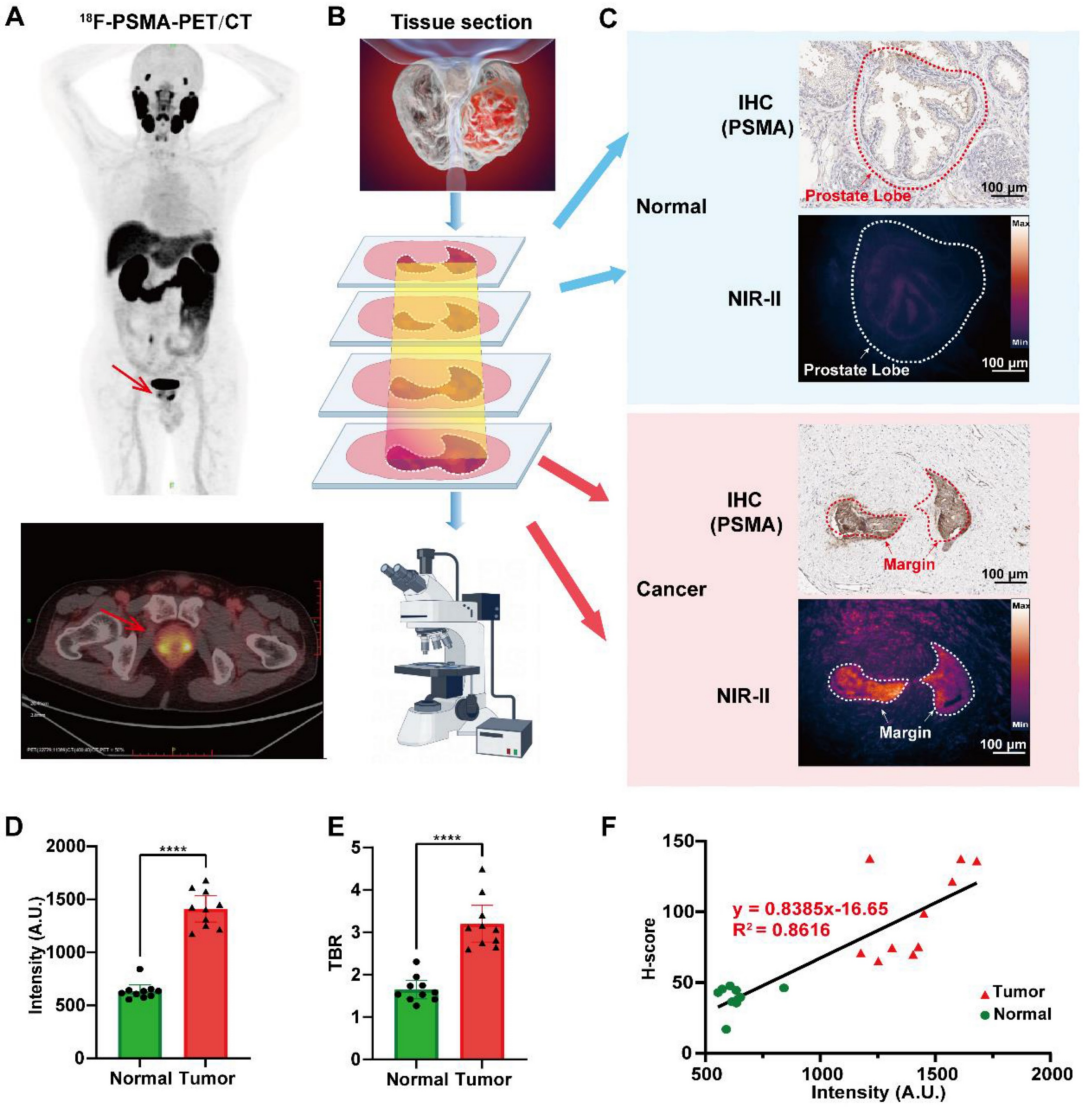
** NIR-II fluorescence imaging enables micron-level margin visualization in clinical PCa specimens.** (A) A 68-year-old male patient with elevated serum PSA (18.06 ng/mL) underwent ^18^F-PSMA PET-CT prior to radical prostatectomy. (B) Resected prostate tissue sections were evaluated by NIR-II fluorescence imaging, PSMA IHC, DAPI nuclear staining, and H&E staining. (C) NIR-II fluorescence microscopy shows micron-level margin visualization with spatial concordance to PSMA IHC (note that fluorescence and IHC are obtained from adjacent sections). (D) Quantitative analysis showing higher fluorescence intensity in tumor tissues than in adjacent normal tissues. (E) TBRs quantified in PSMA-high versus adjacent regions. (F) Correlation between fluorescence intensity and PSMA IHC H-scores across 20 tumor samples from 10 patients (R² = 0.8616, ****P < 0.0001).

To assess the feasibility of histopathological margin mapping, PSMA-12-IRDye800CW was evaluated in FFPE PCa specimens obtained from ten patients undergoing radical prostatectomy, with representative cases localized preoperatively by ¹⁸F-PSMA PET-CT (Fig. 6A). Resected tissues were serially sectioned and examined by NIR-II fluorescence imaging together with matched histopathology, including PSMA IHC, H&E staining, and DAPI nuclear staining (Fig. 6B). In representative sections, NIR-II fluorescence signals were preferentially enriched in tumor regions, whereas adjacent benign regions displayed markedly lower fluorescence (Fig. 6C; Fig. S10).

Notably, NIR-II fluorescence imaging and PSMA IHC were performed on adjacent (not identical) sections from the same FFPE tissue block (Fig. 6C; Fig. S10). Therefore, minor spatial discrepancies—particularly at the tumor-benign interface—are expected due to section-to-section variation, and boundary-width differences between fluorescence and IHC should not be overinterpreted as perfect one-to-one histological equivalence. Within this cautious framework, quantitative analyses demonstrated significantly higher fluorescence intensity in tumor regions than in adjacent normal regions (P < 0.0001; Fig. 6D), accompanied by elevated TBRs in PSMA-high areas (Fig. 6E). Importantly, fluorescence intensity strongly correlated with PSMA IHC H-scores across 20 tumor samples from 10 patients (R² = 0.8616, P < 0.0001; Fig. 6F), supporting a robust relationship between optical signal readouts and target expression.

Collectively, these results indicate that PSMA-12-IRDye800CW enables micron-scale, pathology-aligned fluorescence mapping in clinical prostate specimens and provides a quantitative, PSMA-anchored readout for histopathological margin mapping, while appropriately acknowledging the inherent limitations of adjacent-section comparisons (Fig. 6; Fig. S10).

### Cellular, *in vivo*, and *ex vivo* biosafety assessment of PSMA-12-IRDye800CW

To comprehensively evaluate the biosafety profile of PSMA-12-IRDye800CW, we assessed cytocompatibility *in vitro*, systemic toxicity in mice, and potential immunogenicity using *ex vivo* human BAT.

**Figure 7:**
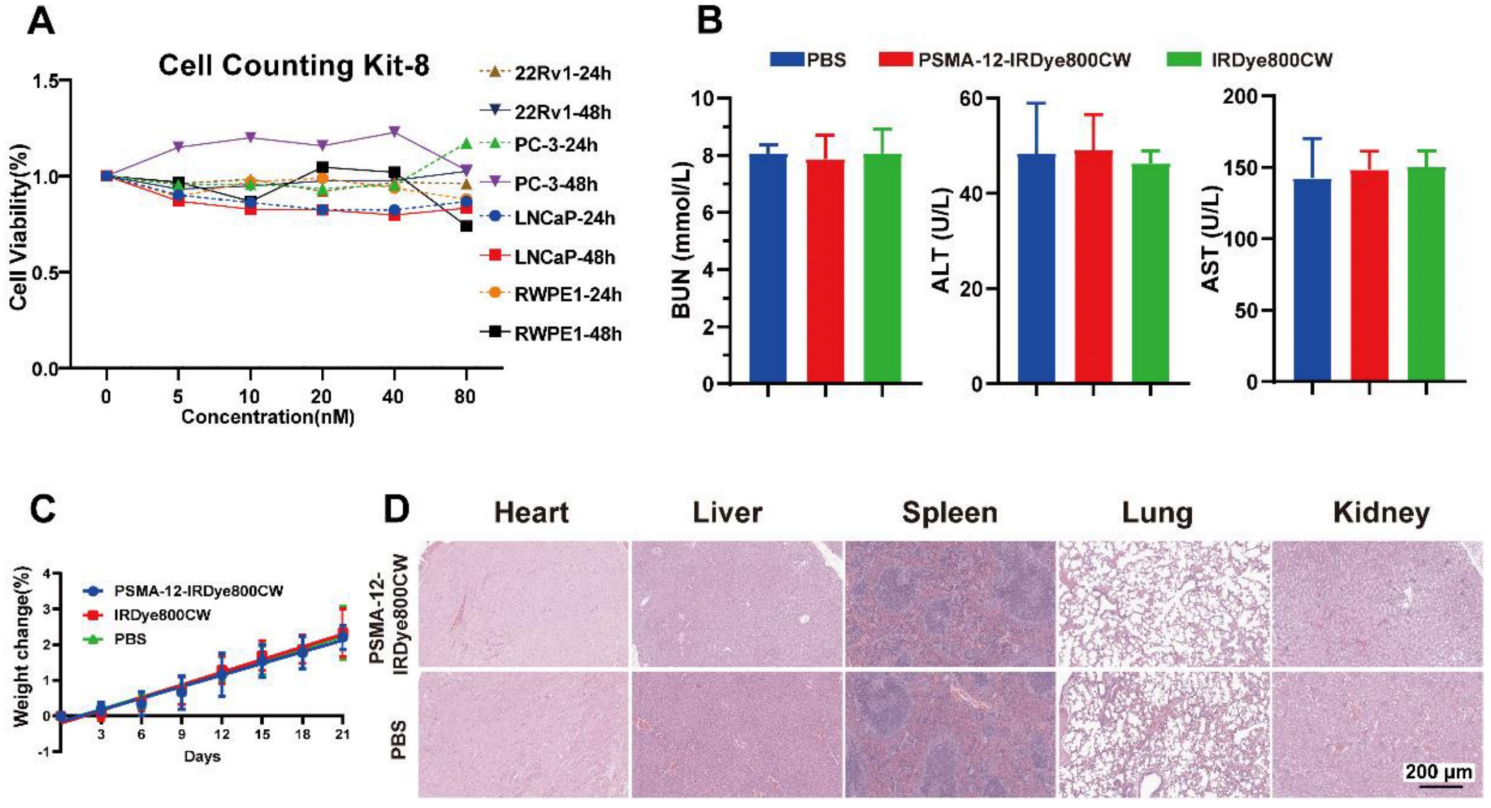
** Comprehensive biosafety evaluation of PSMA-12-IRDye800CW in cellular, murine, and *ex vivo* human serum models.** (A) Cell viability in LNCaP, 22Rv1, PC-3, VCaP, and RWPE-1 cells assessed by CCK-8 assays after 24 h and 48 h incubation with PSMA-12-IRDye800CW (0-80 nM). (B) Mice received intravenous injections of PSMA-12-IRDye800CW, equimolar IRDye800CW, or PBS; renal and hepatic function was assessed at 72 h post-injection by serum BUN, ALT, and AST (n = 5 per group). (C) Body weight monitored every two days for 21 days to evaluate long-term systemic effects (n = 5 per group). (D) H&E staining of major organs (heart, liver, spleen, lungs, kidneys) at 14 days post-injection showing no overt histopathological abnormalities (n = 5 per group). Scale bar = 200 μm.

At the cellular level, CCK-8 assays were performed in LNCaP, 22Rv1, PC-3, VCaP, and RWPE-1 cells after incubation with PSMA-12-IRDye800CW for 24 and 48 h. Cell viability remained comparable to controls across all concentrations and time points, indicating good cytocompatibility (Fig. 7A).

For *in vivo* safety evaluation, mice received intravenous injections of PSMA-12-IRDye800CW, equimolar IRDye800CW, or PBS (n = 5 per group). Renal and hepatic function was assessed at 72 h post-injection by measuring BUN, ALT, and AST, with no significant differences observed among groups (Fig. 7B). Body weight monitoring over 21 days showed no abnormal loss or treatment-related trends (Fig. 7C). Consistently, H&E staining of major organs (heart, liver, spleen, lungs, and kidneys) collected at 14 days post-injection revealed no overt histopathological abnormalities (Fig. 7D).

To estimate the risk of acute hypersensitivity in humans, BAT was conducted using peripheral blood from three healthy donors. PSMA-12-IRDye800CW yielded an SI well below the threshold, indicating no stimulatory effect, while the positive controls N-formylmethionyl-leucyl-phenylalanine (fMLP) and anti-FcεRI produced robust responses (SI = 15.23 and 3.18, respectively) (Table 1).

In addition, IRDye800CW has been evaluated in multiple human studies with favorable safety profiles 43-47. Collectively, these results support the overall biocompatibility of PSMA-12-IRDye800CW and its suitability for further translational evaluation in fluorescence-based molecular imaging.

## Discussion

This study confirmed that PSMA (*FOLH1*) is significantly upregulated from normal prostate tissue to primary PCa and bulk CRPC at the gene, transcript, and protein levels, based on a public transcriptomic cohort and an independent clinical IHC cohort. Notably, a subtype-stratified analysis further showed reduced *FOLH1* expression in late-stage CRPC variants including NEPC and DNPC (Fig. S1), highlighting the molecular heterogeneity of advanced disease. As a clinically validated biomarker and target, PSMA has been widely implemented for molecular imaging and RLT in nuclear medicine 48. PSMA-PET (e.g., ⁶⁸Ga-PSMA-11) is recommended in contemporary clinical guidelines for staging and localization, and PSMA-RLT (e.g., ¹⁷⁷Lu-PSMA-617) has improved outcomes in advanced CRPC 48-50. Building on the clinical relevance of PSMA targeting, we developed PSMA-12-IRDye800CW, a PSMA-617-derived small-molecule fluorescent probe incorporating an albumin-interaction linker to modulate circulation behavior and tumor accumulation, together with PSMA-mediated active targeting 30, 31. The measurable long-wavelength emission tail of IRDye800CW enables fluorescence imaging in the NIR-II window when collected with a 1000 nm long-pass filter, and under matched imaging conditions it provided a quantifiable improvement in spatial resolvability (FWHM: NIR-I 1.06 mm vs. NIR-II 0.93 mm) together with improved depth-dependent structure separability in a tissue phantom model 13, 51 (Fig. 3).

While numerous studies have demonstrated the utility of PSMA-targeted fluorescence agents for PCa surgery, most existing probes, including YC-27, OTL78, and ^68^Ga-P3, primarily operate in the NIR-I window 18, 32-38. These agents have shown promising results in tumor detection and surgical guidance, especially in improving tumor-to-background contrast and aiding in minimizing positive surgical margins. However, the NIR-I window is still limited by significant scattering and autofluorescence, particularly when imaging deeper tissues or complex anatomical structures such as the prostate 18, 32-38. This limitation hinders accurate tumor margin delineation, which is crucial for achieving complete tumor resection during surgery.

Our study addresses this critical gap by shifting to NIR-II fluorescence imaging, which has demonstrated clear advantages in reducing photon scattering and tissue autofluorescence. By utilizing PSMA-12-IRDye800CW, we achieved superior depth penetration and contrast, making it a more effective tool for real-time tumor visualization, particularly in the prostate, where precise margin detection is challenging. This approach marks a significant step forward from the traditional NIR-I agents, offering improved imaging of tumor regions that were previously difficult to visualize with conventional fluorescence-guided systems.

In our *in vivo* 22Rv1 xenograft model, PSMA-12-IRDye800CW demonstrated significantly higher tumor-to-background contrast compared to ICG, including at 24 hours post-injection (TBR: 4.31 ± 0.17 vs. 2.65 ± 0.15). This highlights the extended fluorescence signal retention, which supports an optimized window for fluorescence-guided resection, ensuring more accurate excision of PCa tissue during surgery (Fig. 5B; Fig. S9; Movie 1). Additionally, our findings from human FFPE prostate specimens showed that NIR-II fluorescence signals spatially correlated with PSMA IHC patterns, with a strong correlation between fluorescence intensity and PSMA H-scores (R² = 0.8616). This robust correlation further supports the utility of PSMA-12-IRDye800CW for micron-scale histopathological margin mapping, demonstrating its potential for clinical applications in precise tumor boundary assessment (Fig. 6).

PSMA is a clinically validated target in PCa; however, relying on a single targeting mechanism has practical limitations in real-world applications. For fluorescent probes, sufficiently prolonged *in vivo* circulation time is crucial to enable effective targeting. Albumin-interaction strategies are widely used to prolong circulation and modulate biodistribution, which can help sustain tumor-associated signals; at the same time, albumin-assisted distribution may introduce tissue-context-dependent background arising from permeability, lymphatic drainage, or inflammation-related vascular changes, necessitating cautious interpretation of fluorescence enrichment outside the primary tumor 30, 52-54. Accordingly, PSMA-12-IRDye800CW incorporates a hydrophobic long-chain diacid linker intended to promote noncovalent albumin interaction and thereby extend the practical imaging window. Future studies integrating dedicated albumin-binding assays and pharmacokinetic modeling will be important to quantify the contribution of this module to circulation time, tumor retention, and background dynamics.

In addition, acquisition in the NIR-II window can reduce scattering-related background and improve interpretability under matched imaging settings, which may increase robustness for surgical navigation compared with conventional NIR-I readouts. Prospective validation under standardized operating-room illumination and workflow constraints will be essential to define the practical benefit in real surgical environments 55. Together, these features support the feasibility of fluorescence-guided prostate surgery by improving contrast and visual interpretability during navigation, particularly when precise delineation of tumor-adjacent regions is required.

A key translational aspect of this study is the extension of PSMA-aligned fluorescence readouts from intraoperative navigation to histopathology-oriented evaluation in human FFPE tissues. Complementing conventional H&E-based evaluation, this approach adds a quantitative fluorescence layer that reflects PSMA-associated molecular information and can be cross-referenced with IHC. In FFPE prostate specimens, fluorescence patterns showed spatial correspondence to PSMA IHC on adjacent sections and correlated with PSMA H-scores (R² = 0.8616; Fig. 6). As fluorescence imaging and IHC were performed on adjacent rather than identical sections, slight variations in boundary width may arise due to section-to-section offsets and optical point-spread effects. However, these differences do not affect the ability of specific fluorescence imaging to visually map tumor boundaries at the micron level. These findings suggest that PSMA-12-IRDye800CW may support not only intraoperative navigation but also pathology-aligned assessment of margin-adjacent regions and spatial heterogeneity of PSMA expression within resected tissues. The combination of micron-scale spatial readouts and quantitative correlation with PSMA staining points to a potential role in digital pathology augmentation, contingent on rigorous workflow standardization, imaging calibration, and section-matching strategies.

More broadly, integrating molecular imaging with downstream pathological confirmation aligns with emerging efforts to build a surgery-to-pathology evidence chain for margin assessment and decision support 43, 56-62. Such integration leverages molecularly informed imaging to assist real-time navigation while enabling post hoc interpretation of margin-region findings, thereby bridging macroscopic visualization with microscopic validation 63-65. Lymph node dissection in PCa remains one of the most debated issues in clinical practice 66. In this study, fluorescence-positive lymph nodes detected during FGS (Fig. S9; Movie s1) may represent lymph nodes with metastatic involvement, but could also result from nonspecific accumulation associated with permeability, lymphatic drainage, or inflammation-related vascular changes. Fig. S9 not only confirms the presence of PSMA-positive tumor in resected specimens through H&E and PSMA IHC, but also identifies weak fluorescence signals and confirms them as PSMA-positive micro-metastatic lymph nodes. However, due to experimental limitations, we refrain from drawing definitive conclusions at this time. These observations highlight the importance of pairing fluorescence imaging findings with confirmatory pathology (H&E and PSMA IHC, as shown in Fig. S9) and encourage further validation in metastatic models, along with enhanced nodal workup and standardized criteria for interpreting fluorescence-positive lymph nodes. If lymphatic micro-metastasis can be visualized, it could provide new evidence for refining PCa lymph node dissection paradigms.

Safety remains a cornerstone for clinical translation. PSMA-12-IRDye800CW showed favorable tolerability in mice, with no evident hepatic/renal toxicity markers and no overt histopathological abnormalities (Fig. 7). BAT results were well below commonly used thresholds for hypersensitivity screening, suggesting low acute immunogenic potential under the tested conditions; mechanistic attribution (e.g., albumin interaction) requires further validation 30, 53, 67. These findings, together with the use of clinically deployed dye chemistry and comprehensive characterization (Fig. S3-S8), support further translational evaluation of this PSMA-targeted strategy.

Nonetheless, several limitations should be acknowledged. First, FFPE specimens were used for *ex vivo* mapping to align with routine pathology workflows; evaluation on fresh tissues and prospective intraoperative specimens will be necessary to define practical clinical operating procedures. Second, fluorescence-positive lymph nodes observed in xenograft surgery require validation in clinically relevant metastatic and orthotopic models, together with standardized nodal pathology workup to clarify specificity. Future studies will focus on (i) quantitative albumin-binding and pharmacokinetic profiling, (ii) standardized operating-room illumination validation, and (iii) metastatic-model testing to refine specificity for both intraoperative navigation and pathology-aligned margin mapping.

## Conclusions

In summary, we developed PSMA-12-IRDye800CW, a PSMA-targeted fluorescent probe that integrates PSMA-directed targeting with an albumin-interaction module to support sustained tumor-associated signals. Using 1000 nm NIR-II detection, the probe improved resolvability versus NIR-I under matched conditions, achieved higher *in vivo* tumor-to-background contrast than ICG at defined time points, and enabled PSMA-aligned fluorescence readouts in human FFPE specimens with strong correlation to IHC H-scores. Together, these results support further translational evaluation for fluorescence-guided PCa surgery and histopathological margin mapping.

## Supplementary Material

Additional experimental protocols, extended imaging data, clinical sample details, and supplementary figures and tables.

Supplementary movie.

## Figures and Tables

**Table 1:** Basophil Activation Test for PSMA-12-IRDye800CW.

Group	Sample 1	Sample 2	Sample 3	Avg	SI=Avg/Background
**Background**	2.18	2.33	2.18	2.23	1.00
**PSMA-12-IRDye800CW**	0.48	0.30	1.31	0.70	0.31
**fMLP**	26.70	2.72	72.50	33.97	15.23
**anti-FcεR**	5.23	10.60	5.43	7.09	3.18

**Note**: SI = stimulation index. fMLP = N-formylmethionyl-leucyl-phenylalanine. SI ≥ 2 indicates potential hypersensitivity.

## Data Availability

All data supporting the findings of this study are included in the main text and the Supplementary Information. Additional data are available from the corresponding author upon reasonable request.

## Abbreviations

ALT: alanine aminotransferase
AST: aspartate aminotransferase
BAT: basophil activation test
BSA: bovine serum albumin
BUN: blood urea nitrogen
CCK-8: Cell Counting Kit-8
CRPC: castration-resistant prostate cancer
DNPC: double-negative prostate cancer
DMSO: dimethyl sulfoxide
EPR: enhanced permeability and retention
FGS: fluorescence-guided surgery
fMLP: N-formylmethionyl-leucyl-phenylalanine
FWHM: full width at half maximum
H&E: hematoxylin and eosin
HSA: human serum albumin
ICG: indocyanine green
IHC: immunohistochemistry
Kd: dissociation constant
MFI: mean fluorescence intensity
MW: molecular weight
NEPC: neuroendocrine prostate cancer
NIR: near-infrared
PBS: phosphate-buffered saline
PCa: prostate cancer
PET-CT: positron emission tomography-computed tomography
PSA: prostate-specific antigen
PSMA: prostate-specific membrane antigen
ROI: region of interest
RLT: radioligand therapy
SBR: signal-to-background ratio
SI: stimulation index
TBR: tumor-to-background ratio
TNM: tumor-node-metastasis
