# Supplementary material for: PSMA-targeted fluorescent probe for NIR-II imaging in prostate cancer intraoperative navigation and tumor margin mapping
Source: Theranostics. 2026 Feb 11;16(9):4551–65. doi: 10.7150/thno.117540 (PMC12964127; doi:10.7150/thno.117540)
Supplement: Supplementary file 1 — Additional experimental protocols, extended imaging data, clinical sample details, and supplementary figures and tables. [file thnov16p4551s1.pdf]

Supplementary materials

PSMA-targeted fluorescent probe for NIR-II imaging in prostate cancer intraoperative navigation and tumor margin mapping

Zhongji Jiang[1, 2], Haozhe Tan[3], Bo Wu[4], Lin Zhang[4], Gaohaer Kadeerhan[5], Jin Zhang[4], Jiali Jin[4], Zikuan Zhang[4], Hong Guo[4], Wenmin Guo[2], Jiedong Jia[2], Jun Tian[2], Ben Zhong Tang[3]✉, Dongwen Wang[2, 6]✉

1. Department of Biology, School of Medicine, Southern University of Science and Technology, Shenzhen, Guangdong Province, 518055, China.

2. Department of Urology, National Cancer Center/National Clinical Research Center for Cancer/Cancer Hospital & Shenzhen Hospital, Chinese Academy of Medical Sciences and Peking Union Medical College, Shenzhen, Guangdong Province, 518116, China.

3. Guangdong Basic Research Center of Excellence for Aggregate Science, School of Science and Engineering, The Chinese University of Hong Kong (Shenzhen), Shenzhen, Guangdong Province, 518172, China.

4. Department of Urology, First Hospital of Shanxi Medical University, Taiyuan, Shanxi Province, 030001, China.

5. Central Laboratory & Shenzhen Key Laboratory of Epigenetics and Precision Medicine for Cancers, National Cancer Center/National Clinical Research Center for Cancer/Cancer Hospital and Shenzhen Hospital, Chinese Academy of Medical Sciences and Peking Union Medical College, Shenzhen, Guangdong Province, 518116, China.

6. School of Medicine, Southern University of Science and Technology, Shenzhen, Guangdong Province, 518116, China.

✉Correspondence author: tangbenz@cuhk.edu.cn; urology2007@126.com

Materials

IRDye800CW-NHS ester was purchased from LI-COR Biosciences (USA). Dimethyl sulfoxide (DMSO) was obtained from Solarbio (Beijing, China). Fmoc-Lys(Dde)-Wang resin (0.3–0.8 mmol/g, 100–200 mesh), Fmoc-2-Nal-OH, Fmoc-trans-4-aminomethylcyclohexanecarboxylic acid, 1,18-octadecanedioic acid, and other reagents for solid-phase peptide synthesis (SPPS) were purchased from Nanjing Peptide Industry Biotechnology Co., Ltd. (Nanjing, China). DIPEA, HOBt, and DIC were obtained from standard commercial suppliers. The PSMA/GCPII polyclonal antibody was purchased from Proteintech (Wuhan, China). Male BALB/c mice (4–6 weeks old) were supplied by GemPharmatech (Suzhou, China).

Synthesis of PSMA-12 (Compound 6)

PSMA-12 (Compound 6) was synthesized via stepwise Fmoc-SPPS on Fmoc-Lys(Dde)-Wang resin (Compound 1) following the route shown in Fig. S2. Briefly, the resin was swollen in DMF and the N-terminal Fmoc group was removed using 25% piperidine in DMF (v/v) to afford a free amine. For the synthesis of Compound 2, H-Glu(OtBu)-OtBu was coupled onto the deprotected resin using N,N′-disuccinimidyl carbonate in the presence of DIPEA in DMF. The Lys side-chain Dde group was selectively removed using 2% hydrazine in DMF (v/v), followed by coupling of Fmoc-2-Nal-OH to obtain Compound 3. After Fmoc deprotection (25% piperidine/DMF), Fmoc-trans-4-

aminomethylcyclohexanecarboxylic acid was coupled to yield Compound 4. Subsequent Fmoc deprotection enabled coupling of an Fmoc-protected Lys derivative to afford Compound 5. After selective removal of the Lys side-chain Dde group (2% hydrazine/DMF), 1,18-octadecanedioic acid (10 equiv) was coupled to introduce the long-chain diacid linker, followed by final Fmoc deprotection to complete the assembly of PSMA-12 on resin. The fully assembled product (Compound 6) was cleaved from the resin and side-chain protecting groups were removed using a cleavage cocktail of TFA/H_2O/TIPS (90:5:5, v/v/v) at 30 °C for 3 h. The filtrate was concentrated and precipitated in cold anhydrous diethyl ether, and the crude product was collected by centrifugation, washed with ether, and dried. PSMA-12 was purified by preparative reversed-phase HPLC and lyophilized to yield the final intermediate (Fig. S2–S7).

Synthesis of PSMA-12-IRDye800CW

PSMA-12-IRDye800CW was prepared by conjugating PSMA-12 (Compound 6) with IRDye800CW-NHS ester (Fig. S2). PSMA-12 was dissolved in water (0.01 mg/μL), followed by addition of Na_2HPO_4 buffer (0.01 M, pH 8.5). IRDye800CW-NHS (1.0 equiv) was dissolved in DMSO and added dropwise to the peptide solution under stirring at room temperature for 2 h. The crude conjugate was purified by preparative RP-HPLC and lyophilized to obtain PSMA-12-IRDye800CW. The chemical structure and molecular weight of PSMA-12-IRDye800CW are shown in Fig. S8. The final compound had a molecular formula of $C_{103}H_{141}N_9O_{27}S_4$ and a molecular weight of 2065.54 g/mol.

Synthesis of PSMA-12-FITC

PSMA-12-FITC was synthesized using the same PSMA-12 intermediate. Briefly, PSMA-12 was reacted with FITC under basic aqueous conditions to afford the fluorescein-labeled analog, followed by preparative RP-HPLC purification and lyophilization. The chemical structure and molecular weight of PSMA-12-FITC are shown in Fig. S8. Its molecular formula is $C_{78}H_{100}N_8O_{18}S$ with a molecular weight of 1469.76 g/mol and excitation/emission maxima of 495/519 nm.

Analytical characterization

Analytical HPLC was performed on a reversed-phase C18 column (4.6 mm × 250 mm, 5 μm) using water (A, 0.1% TFA) and acetonitrile (B, 0.1% TFA) as the mobile phases with a linear gradient of 5–95% B at 1.0 mL/min; detection was typically monitored at 220 nm for peptide intermediates (Fig. S4) and at appropriate wavelengths for dye-labeled conjugates. The purity of PSMA-12 was determined to be 98.53% by analytical HPLC (Fig. S4). MALDI-TOF mass spectrometry was used to confirm the molecular identity of PSMA-12 (Fig. S5) on a MALDI-TOF instrument ([model], [manufacturer]) using a suitable matrix (e.g., sinapinic acid) in positive-ion mode. For NMR analysis, purified PSMA-12 was dissolved in DMSO-d_6 and recorded on a Bruker Avance III 600 MHz spectrometer (Bruker, Germany) at 25 °C to obtain ^1H NMR (Fig. S6) and ^{13}C NMR (Fig. S7), confirming characteristic resonances consistent with the PSMA-12 scaffold and linker. Optical density (OD) measurements were performed using a NanoDrop 2000 spectrophotometer (Thermo Scientific, USA). UV–vis absorption spectra were acquired using a UV-2600 spectrophotometer (Hitachi High-Technologies, Japan).

Fluorescence excitation and emission spectra were recorded on an FLS1000 steady-state/transient spectrofluorometer (Edinburgh Instruments, UK). For PSMA-12-IRDye800CW, OD values at 290 nm and 808 nm were used to establish standard calibration curves and to calculate the dye-to-probe ratio (F/P) based on linear regression.

80 Supplementary figures

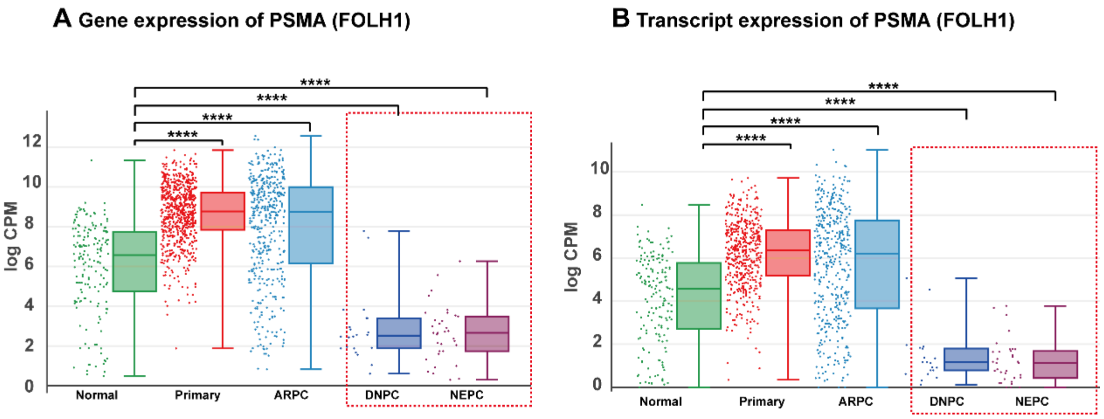

Figure S1. Expression of PSMA (*FOLH1*) across prostate cancer stages. (A) Gene-level and (B) transcript-level expression of PSMA (*FOLH1*) in normal prostate tissue (n = 173), primary prostate cancer (n = 708), castration-resistant prostate cancer (CRPC, n = 428), neuroendocrine prostate cancer (NEPC, n = 34), and double-negative prostate cancer (DNPC, n = 22). Statistical analysis was performed using unpaired two-tailed t-tests; ****P < 0.0001 for all comparisons versus normal tissue.

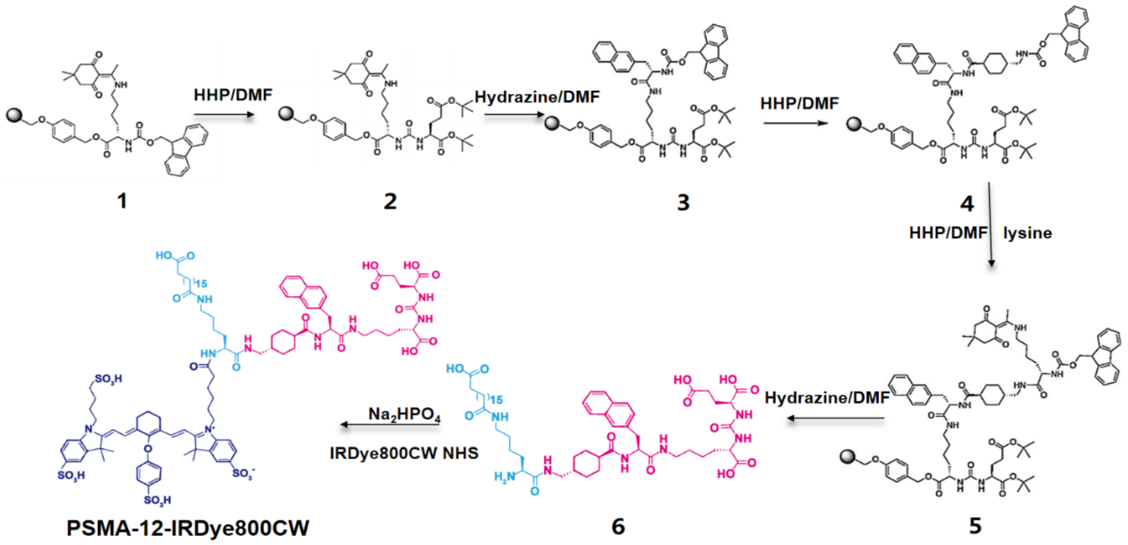

Figure S2. Synthetic route of the PSMA-12-IRDye800CW dual-targeted fluorescent probe. (1) A resin-bound Glu-urea intermediate was extended through sequential coupling and deprotection reactions. (2–4) A lysine residue and an α,ω-dicarboxylic acid linker were introduced to enable albumin interaction. (5–6) Final conjugation of the peptide intermediate with IRDye800CW NHS ester was performed under aqueous basic conditions (Na_2HPO_4), followed by HPLC purification. The resulting PSMA-12-IRDye800CW probe comprises a Glu-urea-Lys targeting motif, an albumin-interaction linker, and IRDye800CW to enable fluorescence imaging, including NIR-II imaging capability under a 1000 nm long-pass detection window.

81

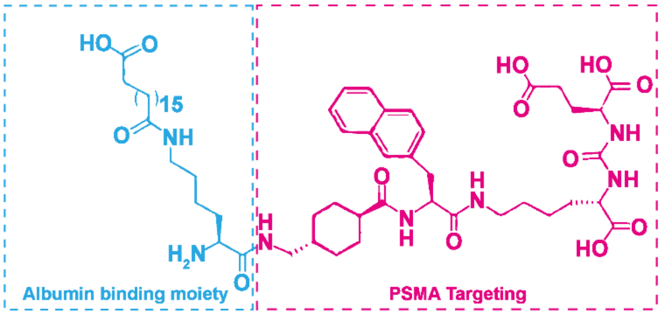

Figure S3. Chemical structure and molecular weight of the key synthetic intermediate PSMA-12 (Compound 6). Chemical structure of Compound 6 (PSMA-12), the critical peptide intermediate used for subsequent conjugation with IRDye800CW; calculated and experimentally determined molecular weights are provided.

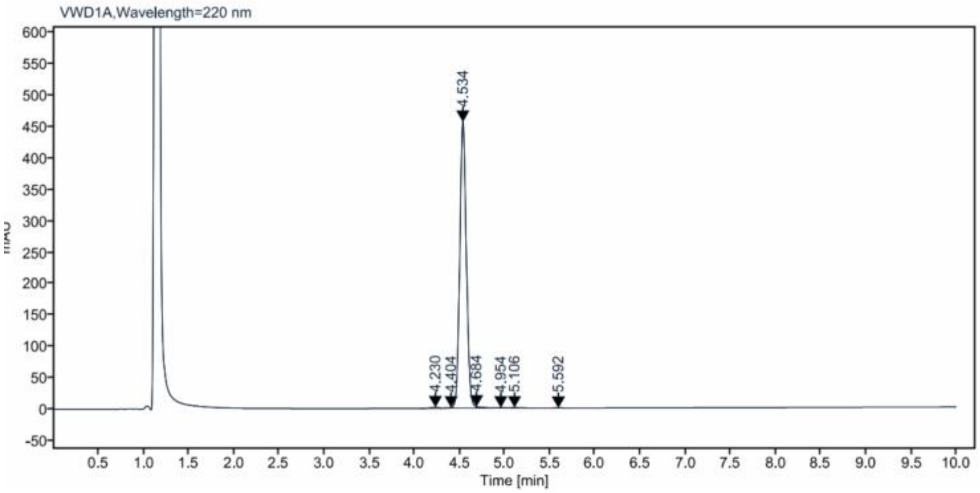

Signal: VWD1A,Wavelength=220 nm

RT [min]	Type	Width [min]	Height	Area	Area%
4.230	BV	0.241	1.600	9.905	0.423
4.404	VV	0.099	1.096	3.379	0.144
4.534	VF	0.280	456.534	2306.380	98.534
4.684	VV	0.183	2.337	11.176	0.477
4.954	VBA	0.144	0.439	2.364	0.101
5.106	BV	0.294	0.838	5.414	0.231
5.592	BV	0.233	0.419	2.075	0.089

Figure S4. HPLC purity analysis of the PSMA-12 intermediate. Analytical HPLC chromatogram of PSMA-12 showing a purity of 98.53%, supporting its suitability for downstream conjugation.

82

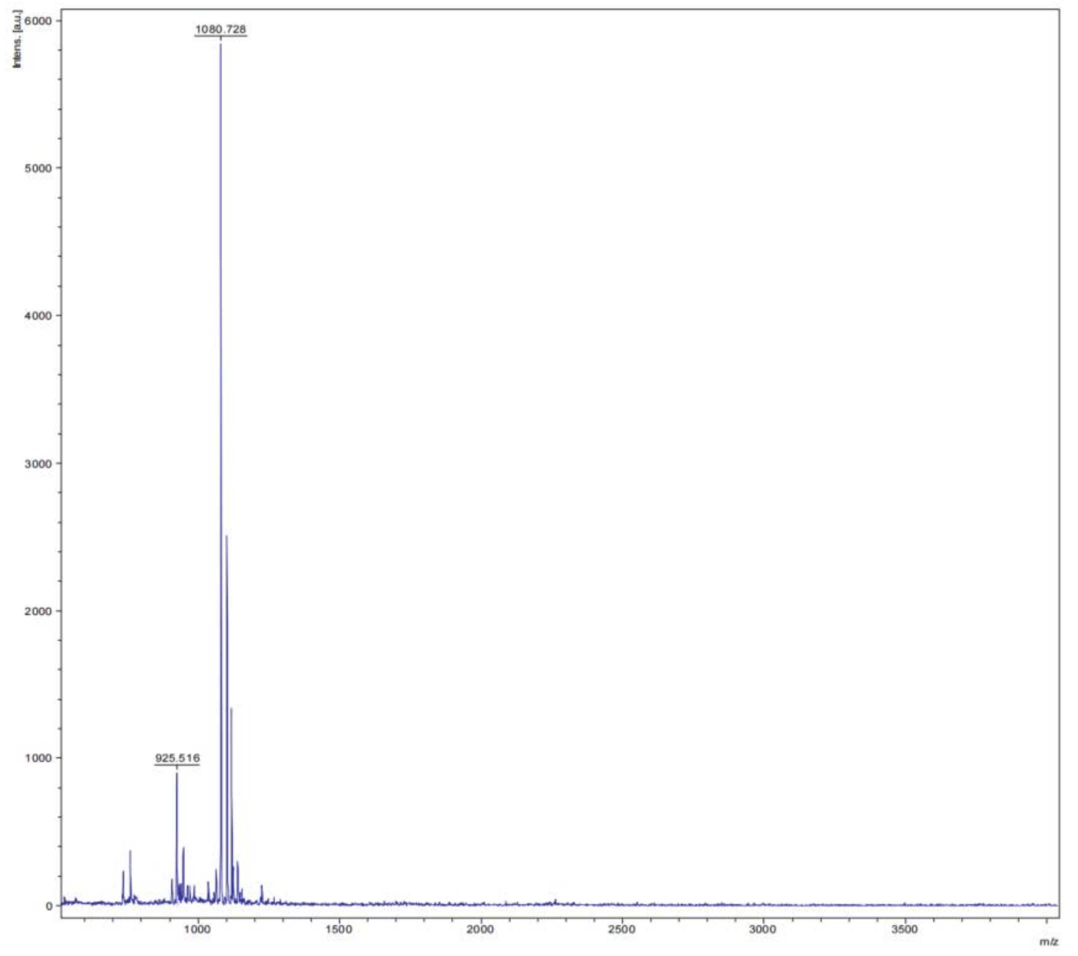

Figure S5. MALDI-TOF mass spectrometry of PSMA-12. MALDI-TOF mass spectrum of PSMA-12 showing a dominant peak corresponding to the expected molecular ion.

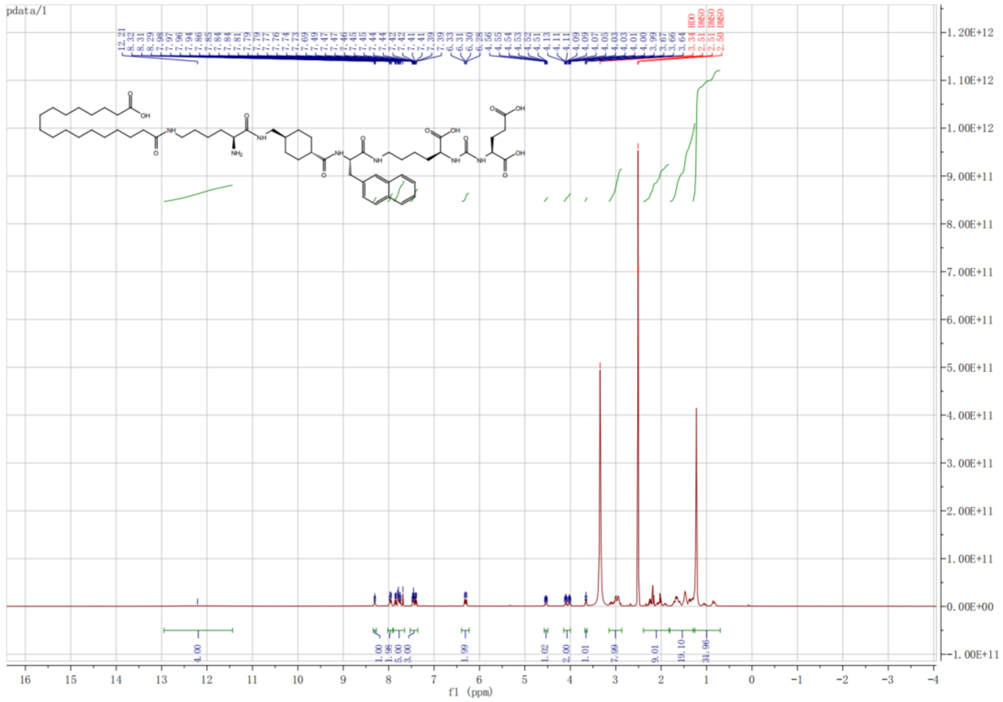

Figure S6. ^{1}H NMR spectrum of PSMA-12. ^{1}H NMR spectrum acquired in DMSO-d_6 confirming characteristic chemical shifts and proton integration patterns consistent with the PSMA-12 scaffold and linker.

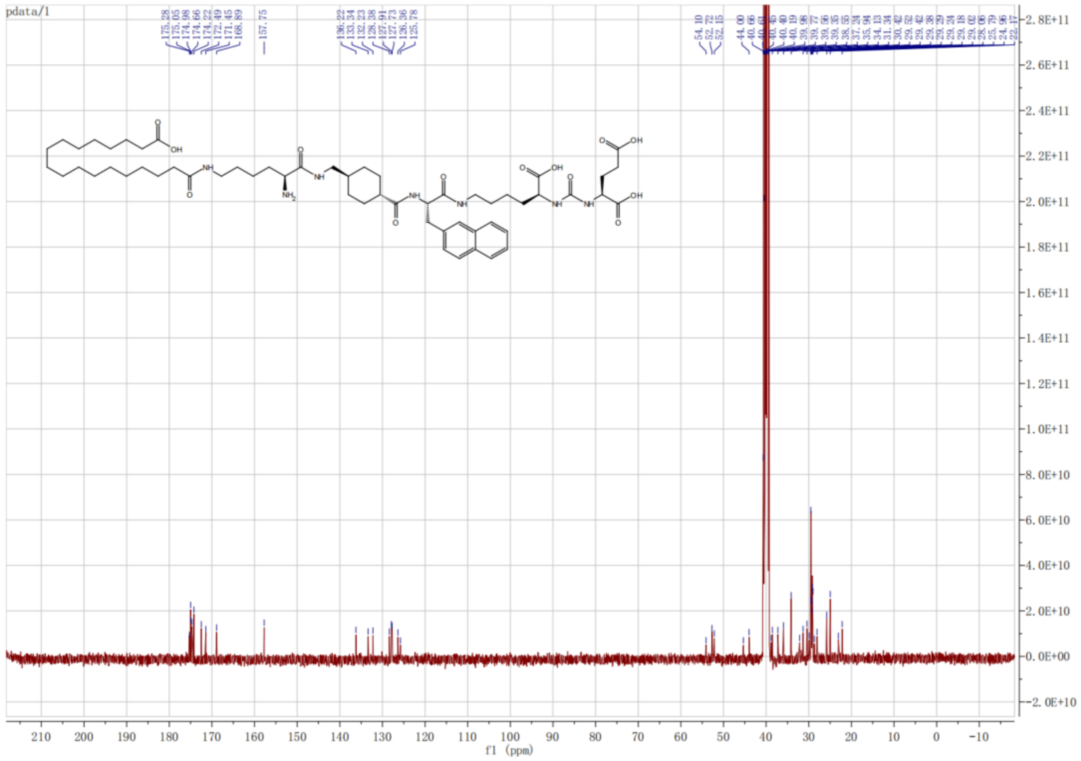

Figure S7. ^{13}C NMR spectrum of PSMA-12. ^{13}C NMR spectrum confirming characteristic carbon resonances of PSMA-12, including carbonyl/urea and aliphatic carbon signals.

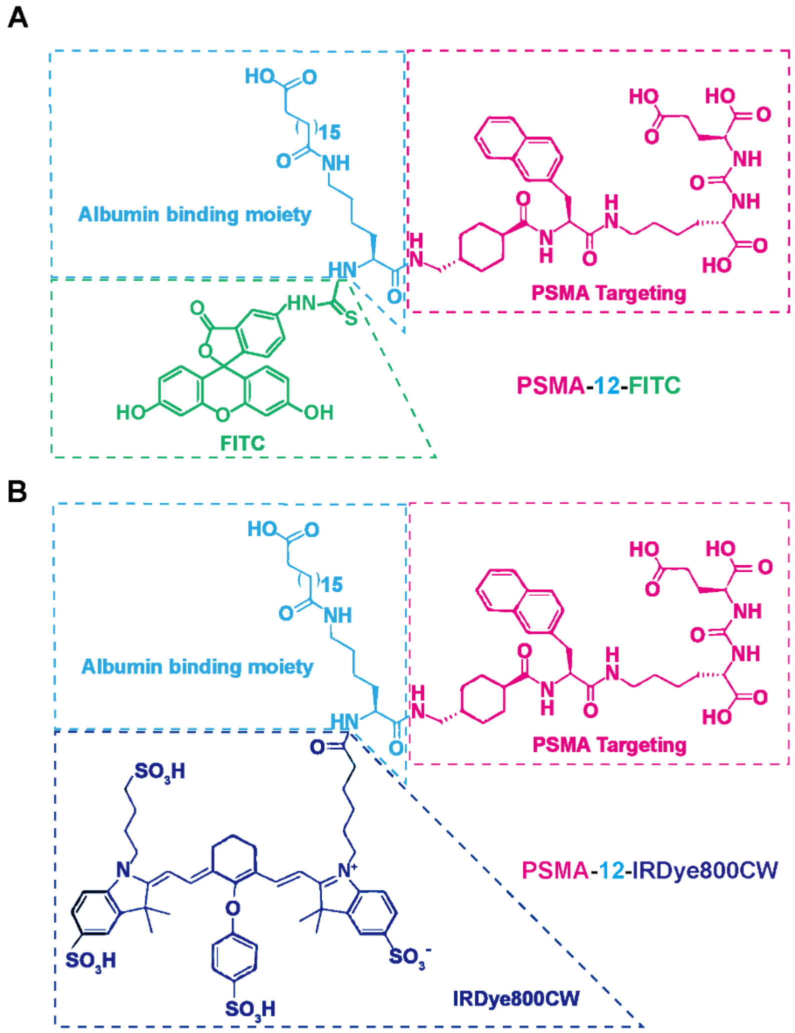

Figure S8. Chemical structures and molecular weights of PSMA-12-FITC and PSMA-12-IRDye800CW. Chemical structures of PSMA-12-FITC (left) and PSMA-12-IRDye800CW (right), each incorporating the Glu-urea-Lys targeting motif and an albumin-interaction linker. PSMA-12-FITC: $C_{78}H_{100}N_9O_{18}S$, 1469.76 Da; PSMA-12-IRDye800CW: $C_{103}H_{149}N_9O_{27}S_4$, 2085.54 Da.

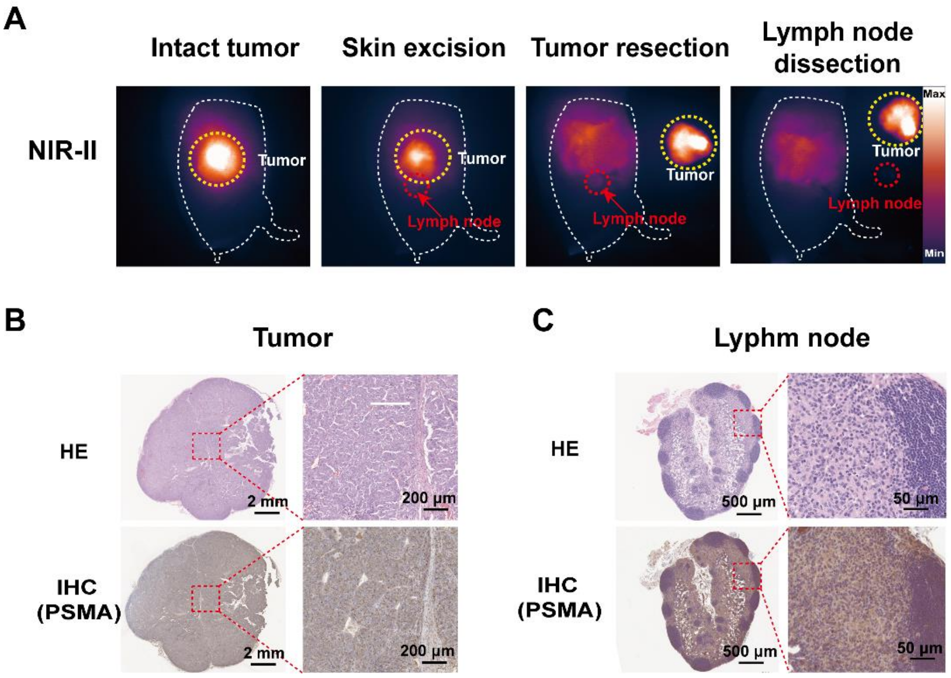

Figure S9. In vivo NIR-II fluorescence-guided surgery using PSMA-12-IRDye800CW. Intraoperative NIR-II fluorescence imaging showing intact tumor visualization, skin incision, fluorescence-guided tumor resection, and identification of fluorescence-positive lymph nodes. Postoperative pathological validation of tumor and resected lymph nodes by H&E staining and PSMA IHC is provided.

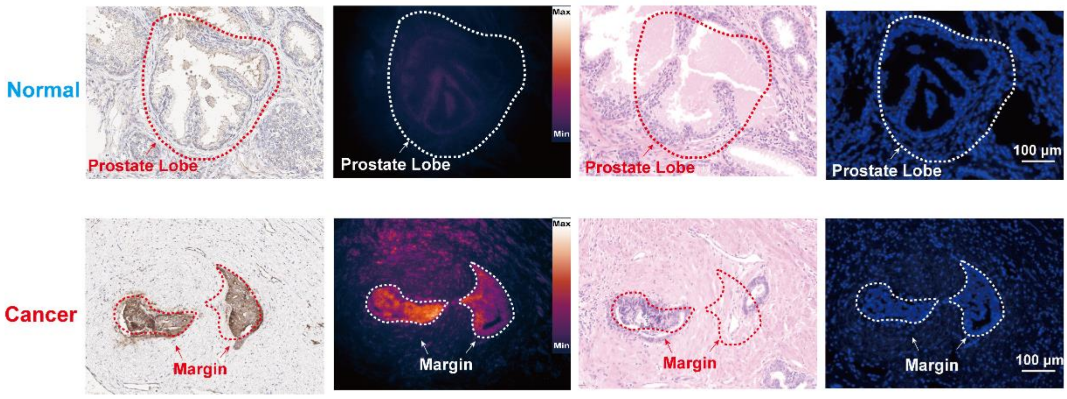

Figure S10. Histopathology-aligned fluorescence mapping of tumor-adjacent regions in human prostate cancer specimens. Representative images of prostate cancer tissue and adjacent benign regions, including PSMA IHC, NIR-II fluorescence imaging, H&E staining, and DAPI nuclear staining. Fluorescence and PSMA IHC were acquired from adjacent (non-identical) serial sections; therefore, modest spatial offsets and apparent boundary-width differences may occur. Despite these section-to-section variations, fluorescence signals show spatial correspondence to PSMA IHC–positive regions, supporting histopathology-aligned margin-region assessment. Scale bar = 100 μm.

Supplementary tables.

Supplementary table 1

**Group**	***FOLH1***	**P value 1**	**Transcripts**	**P value 2**
**Normal**	6.01±2.26		3.76±1.87	
**Primary**	8.68±1.46	＜0.0001	5.68±2.08	＜0.0001
**CRPC**	7.98±2.65	＜0.0001	5.46±2.76	＜0.0001
**NEPC**	2.63±1.41	＜0.0001	4.20±2.66	＜0.0001
**DNPC**	2.87±1.74	＜0.0001	5.47±2.37	＜0.0001

Primary: Primary Prostate Cancer; CRPC: Castration-Resistant Prostate Cancer; NEPC: Neuroendocrine Prostate Cancer; DNPC: Double Negative Prostate Cancer. P value 1: P value of the *FOLH1* gene expression value (vs.Normal); P value 2: P value of the *FOLH1* Transcripts expression of value (vs. Normal).

Supplementary Table 2. Clinical and Pathological Characteristics of Patients with PCa

NO.	Age (years)	tPSA (ng/ml)	Gleason Score	TNM	Metastatic Site	Histopathological Diagnosis
1	64	124.70	4+4	ypT3bN1	Left pelvic lymph nodes	Prostatic acinar adenocarcinoma
2	66	101.50	4+3	ypT3aN1	Right pelvic lymph node	Prostatic acinar adenocarcinoma
3	73	4.10	4+3	pT2aN0M0	-	Prostatic acinar adenocarcinoma
4	63	31.68	4+5	pT3bN0M0	-	Prostatic acinar adenocarcinoma
5	70	28.29	3+4	pT3bN1	Left pelvic lymph nodes	Prostatic acinar adenocarcinoma
6	58	5.66	3+3	pT3bN0	-	Prostatic acinar adenocarcinoma
7	67	11.86	3+4	pT2aN0M0	-	Prostatic acinar adenocarcinoma
8	62	31.21	5+4	ypT2N2M1b	Fifth lumbar vertebra（bone metastasis）	Prostatic acinar adenocarcinoma
9	69	18.06	3+4	pT3aN0M0	-	Prostatic acinar adenocarcinoma
10	63	0.33	4+3	ypT3bN1	Left obturator lymph nodes；Right obturator lymph nodes	Prostatic acinar adenocarcinoma；intraductal carcinoma

TNM: Tumor-Node-Metastasis
